# Mechanisms and biomarkers of poststroke cognitive impairment

**DOI:** 10.4103/NRR.NRR-D-25-00674

**Published:** 2026-01-27

**Authors:** Mengxia Liu, Manqing Zhang, Zhiying Chen, Bing Bao, Yanghang Chen, Fangfang Wang, Min Jiang, Moxin Wu, Xiaoping Yin

**Affiliations:** 1 Department of Medical Laboratory, Affiliated Hospital of Jiujiang University, Jiujiang, Jiangxi Province, China; 2 Department of Neurology, Affiliated Hospital of Jiujiang University, Jiujiang, Jiangxi Province, China; 3 Medical College of Jiujiang University, Jiujiang, Jiangxi Province, China; 4 Jiujiang Clinical Precision Medicine Research Center, Jiujiang, Jiangxi Province, China

**Keywords:** biomarkers, blood–brain barrier, demyelination, inflammasomes, neuroinflammation, neuroimaging, oxidative stress, poststroke cognitive impairment, stroke, white matter

## Abstract

Poststroke cognitive impairment is a common neurological complication in stroke patients, characterized by progressive cognitive decline ranging from mild cognitive impairment to vascular dementia, significantly impacting patients’ quality of life and long-term prognosis. Recent studies have gradually unveiled the multidimensional pathophysiological mechanisms underlying post-stroke cognitive impairment. This review provides a detailed introduction to the mechanisms and biomarkers of poststroke cognitive impairment. At the molecular level, the development of poststroke cognitive impairment involves multi-level and interconnected pathophysiological changes. Among these, the activation of neuroinflammation and oxidative stress damage are considered key initiating factors. Concurrently, the accumulation of reactive oxygen species induced by oxidative stress can further promote the occurrence of poststroke cognitive impairment. Increased blood–brain barrier permeability, along with the infiltration of peripheral inflammatory cells and the entry of toxic substances into the brain, exacerbates neural damage. In terms of neurotransmitter systems, the imbalance between excitatory and inhibitory neurotransmitter systems directly affects synaptic plasticity and the integration of neural networks. Structurally, the integrity of white matter microstructure is compromised, manifesting as myelin loss and axonal transport impairment. These multi-level pathological changes interact through complex positive feedback mechanisms, collectively forming the pathogenic network of poststroke cognitive impairment. In the field of biomarker research, a six-dimensional classification system for post-stroke cognitive impairment biomarkers has been reported, systematically categorizing relevant biomarkers into metabolic markers, inflammatory factor profiles, genetic markers, blood–brain barrier damage indicators, gut microbiota characteristics, and neuroimaging biomarkers. Notably, the integrated predictive model developed by combining serum biomarkers with multimodal neuroimaging features significantly enhances the diagnostic specificity of poststroke cognitive impairment biomarkers. This multidimensional and systematic research approach provides new perspectives for the in-depth analysis of the pathogenesis of poststroke cognitive impairment biomarkers and offers important targets for early clinical intervention. In the future, it is hoped that dynamic monitoring of the temporal changes in these biomarkers can more accurately assess the effectiveness of interventions and guide the optimization and adjustment of treatment plans. Based on risk assessment results, a tiered management approach should be implemented: high-risk patients should undergo cognitive assessments and biomarker testing every three months, while moderate-to-low-risk patients should follow a stepwise monitoring protocol. This precision management model can predict the risk of cognitive decline up to 6–12 months in advance, enabling timely interventions to reduce the incidence of severe cognitive impairment. Mechanistic studies and biomarker discovery for poststroke cognitive impairment have brought breakthrough progress to clinical diagnosis and treatment. Future research should continue to explore its molecular mechanisms, develop more effective targeted therapeutic drugs, establish a comprehensive early warning and personalized treatment system, and ultimately achieve precise prevention, control, and optimized management of poststroke cognitive impairment.

**Facts**

• Multi-mechanism collaborative pathogenic mechanism: The occurrence and progression of poststroke cognitive impairment involve complex interactions of various pathophysiological changes, including neuroinflammation activation, oxidative stress, blood–brain barrier disruption, and abnormal neural network connectivity.

• Multi-group system classification of biomarkers: Biomarkers related to poststroke cognitive impairment encompass multiple dimensions such as metabolism, inflammation, genetics, gut microbiota, and neuroimaging, providing a rich information source for early identification and precise diagnosis.

• Integration of multi-modal data to enhance diagnostic precision: By integrating neuroimaging, serum biomarkers, and clinical data, a multi-dimensional assessment system can be constructed, significantly improving the reliability of early identification and prognosis prediction for poststroke cognitive impairment, paving new pathways for stratified interventions.

**Open questions**

• Is there a clear cascading relationship among the aforementioned pathogenic mechanisms (neuroinflammation, gut microbiota dysbiosis, and blood-brain barrier damage)? Is there a core driving factor that could serve as the most effective therapeutic target?

• Can a high-precision and clinically valuable early prediction and individualized risk stratification model for poststroke cognitive impairment be established based on multi-dimensional biomarkers?

• In light of the identified key mechanisms, how can targeted neuroprotective drugs or intervention strategies be developed to effectively delay or reverse cognitive decline following a stroke?

## Introduction

**Figure 1:**
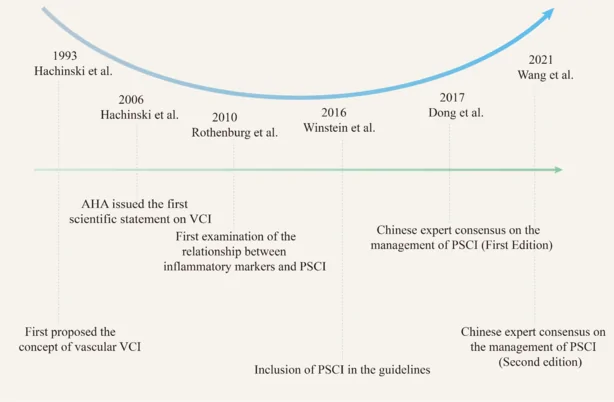
Milestones in PSCI research. This figure presents a timeline highlighting key developmental milestones in the field of poststroke cognitive impairment. The progression demonstrates a shift from the international conceptualization and mechanistic exploration toward the integration into clinical guidelines and the formulation of regional consensus. This evolution underscores the systematic advancement of the field from theoretical foundations to standardized clinical practice. AHA: American Heart Association; PSCI: poststroke cognitive impairment; VCI: vascular cognitive impairment.

Stroke, as the second leading cause of death and the third leading cause of disability worldwide, not only leads to motor and sensory impairments but is also a major cause of vascular cognitive impairment (VCI) (Hachinski and Bowler, 1993; Hachinski et al., 2006). Poststroke cognitive impairment (PSCI), as a manifestation of VCI, has become one of the core issues in stroke rehabilitation and neuro-regeneration research (Winstein et al., 2016). Study indicates that PSCI affects approximately 20%–82% of stroke survivors (Filler et al., 2024), with stroke increasing the risk of cognitive impairment by at least 5-fold and even up to 8-fold (Ashburner et al., 2024). In addition, research has shown that poststroke patients with mild cognitive impairment (MCI) accompanied by apathy have a significantly higher likelihood of progressing to dementia within a 6-month follow-up period. PSCI typically presents with multidomain cognitive impairments, characterized by executive dysfunction, attention deficits, and visuospatial deficits, among which executive dysfunction is the earliest and most important core clinical feature (Sakai et al., 2024). Clinical evidence indicates that PSCI not only adversely affects motor function and daily activities but also complicates rehabilitation, impedes functional recovery, and hinders reintegration into family and societal roles (He et al., 2023; Gallucci et al., 2024). However, the specific pathophysiological mechanisms underlying PSCI remain unclear (**Figure 1**).

Current evidence suggests that neuroinflammation plays a central role. Inflammasome activation triggers the release of proinflammatory cytokines, contributing to neuronal injury and the suppression of neural stem cell (NSC) activity. Concurrently, oxidative stress exacerbates the neurogenic microenvironment through free radical accumulation, leading to reduced NSC levels and pyroptosis. Neurotransmitter imbalances further aggravate cognitive deficits through impaired synaptic plasticity. Additionally, compromised white matter integrity constitutes a critical component of this pathological network. Studies have described a strong correlation between microstructural damage to the white matter tract and executive dysfunction (Hilal et al., 2021; Ribeiro et al., 2024). Disruption of the blood–brain barrier (BBB) facilitates abnormal amyloid-β (Aβ) deposition and the leakage of neurotoxic substances, thereby triggering a secondary neuroinflammatory response. However, current research is largely confined to exploring single mechanisms, lacking an integrated analysis of multi-system interactions. Moreover, most clinical studies suffer from limited sample sizes and short follow-up periods, failing to systematically elucidate the specific mechanisms underlying the occurrence of PSCI. This review aims to systematically integrate the multidimensional pathological mechanisms of PSCI to address research gaps in this field. Building upon this foundation, it innovatively proposes a biomarker classification framework encompassing multiple categories including metabolism, inflammation, genetics, imaging, and the microbiome.

Compared to previous studies, this review not only strives to construct a more comprehensive theoretical framework but also attempts to translate this framework into an integrated biomarker system applicable to clinical practice. The ultimate goal is to enable early warning of PSCI, objective assessment of its severity, and dynamic monitoring of rehabilitation outcomes, thereby providing key strategies and directions for advancing precision prevention, control, and personalized treatment management of PSCI.

## Search Strategy

We searched the PubMed and Web of Science databases for studies published between January 2005 and March 2025. The search strategy incorporated both free-text terms and subject headings, as well as disease-related keywords such as “stroke” and “post-stroke cognitive impairment.” These were combined with mechanism and biomarker terms such as “biomarker,” “neuroinflammation,” “oxidative stress,” “neurotransmitter,” “white matter injury,” and “remyelination.” The inclusion criteria were as follows: all clinical studies evaluating changes in various biomarkers in patients with PSCI were included, while interventional studies and animal experiments were excluded. In this study, we used the Newcastle-Ottawa Scale (NOS) and the QUADAS-2 tool to assess the quality of the literature. The NOS evaluates three dimensions: study subject selection (4 points), intergroup comparability (2 points), and outcome measurement (3 points), with a total score of 7–9 points indicating a high-quality study. The QUADAS-2 tool provides a supplementary assessment based on four dimensions: case selection, detection criteria, the gold standard, and the detection process. Through the combined application of these two evaluation systems, 242 studies meeting high-quality standards were ultimately selected from the included literature. Two researchers (MZ and ZC) conducted independent quality assessments, with discrepancies resolved through discussion with a third researcher (BB).

## Potential Mechanisms of Poststroke Cognitive Impairment

### Neuroinflammation contributes to post-stroke cognitive impairment progression

Neuroinflammation significantly contributes to secondary brain injury in both patients with acute ischemic stroke (IS) and hemorrhagic stroke. The neuroinflammatory response is initiated within minutes of blood flow interruption and may persist for several days, characterized by the coordinated activation of multiple cell types, including central nervous system (CNS)–derived microglia and astrocytes, vascular endothelial cells (ECs), and infiltrating peripheral immune cells. This process is accompanied by the release of proinflammatory cytokines, chemokines, and cell adhesion molecules. Consequently, these processes contribute to long-term poststroke sequelae, including motor deficits and cognitive impairment. Accumulating evidence underscores the critical role of neuroinflammation in mediating neuronal cell death after stroke. Chronic inflammation is closely linked to the pathogenesis of Alzheimer’s disease (AD) and other forms of dementia (Andronie-Cioara et al., 2023). The absent in melanoma 2 (AIM2) inflammasome has garnered significant attention because of its roles in neuronal injury and the regulation of dendritic and axonal growth. Kim et al. (2020a) reported the significant activation of AIM2 inflammasomes following ischemic injury. Their study further revealed that AIM2 activation suppresses hippocampal cell proliferation and induces neuronal cell death, indicating that these pathological mechanisms are involved in the development of PSCI. Similarly, Jiao et al. (2022) used both mouse models and *in vitro* stroke models to confirm that LACC1 aggravates neuroinflammation and oxidative stress by blocking the AMP-activated protein kinase (AMPK) signaling pathway and activating the NOD-like receptor protein 3 (NLRP3) inflammasome, thereby inducing PSCI.

A study has shown that astrocytes are significantly activated after stroke through upregulation of glial fibrillary acidic protein (GFAP) expression and the production of various proinflammatory factors. Huang et al. (2020) identified a correlation between reactive astrocyte proliferation and the severity of PSCI using ^18^F-THK-5351 imaging to assess reactive astrocyte proliferation in amyloid-negative stroke patients. Additionally, a study demonstrated increased 18F-THK-5351 uptake in peri-infarct regions following stroke and attenuation away from the stroke lesion. These scar-forming astrocytes can be used to isolate the nonfunctional and nonneural core of the injury from the surrounding neural tissue, which remains potentially functional. While earlier work indicated the neurotrophic role of astrocyte-derived scaffolding proteins in neuronal pathfinding (Kim and Chung, 2023), recent studies have revealed that glial scar formation is a major obstacle to axonal regeneration in the CNS (Akam et al., 2022; Zeng et al., 2024). Therefore, the disruption of glial–neuronal interactions may contribute to synaptic dysfunction and cognitive decline.

As the predominant resident macrophages in the CNS, microglia act as the first line of defence in the pathological process of stroke. Cerebral ischaemia triggers rapid microglial activation, which amplifies neuroinflammatory cascades through the release of proinflammatory mediators such as tumour necrosis factor-α (TNF-α) and interleukin-1β (IL-1β). Microglia can be polarized into proinflammatory M1 and anti-inflammatory M2 phenotypes, both of which critically regulate neuroinflammatory responses. M1 microglia release proinflammatory cytokines and reactive oxygen species (ROS). In contrast, M2 microglia secrete anti-inflammatory cytokines that promote tissue regeneration, injury repair, and neovascularization. Additionally, they produce insulin-like growth factors (IGFs), which inhibit apoptosis and support neuronal growth and recovery. However, during the acute phase of IS, microglia undergo a phenotypic shift from the M2 phenotype towards the M1 phenotype. This proinflammatory transition aggravates neuronal damage and cell death. A current study indicates that microglia-induced neuronal damage and functional impairment represent core mechanisms underlying the development and progression of PSCI (Zeng et al., 2024). Key signaling pathways (including TLR4, p25/CDK5, NF-κB, and CX3CR1) are collectively involved in regulating this pathological process. These signaling pathways drive M1-type microglial polarization, induce neuroinflammatory responses, and exacerbate neuronal injury. Research has indicated that microglia markedly suppress both the proliferation of neural stem/progenitor cells and their neuronal differentiation, thereby significantly impairing neurogenesis. Nuclear factor kappa-light-chain-enhancer of activated B cells (NF-κB) plays a central regulatory role in canonical signaling pathways. Specifically, high mobility group protein B1 (HMGB1) activates Src kinase to initiate the NF-κB signalling cascade, which drives the polarization of microglia towards the proinflammatory M1 phenotype. This signal transduction process further facilitates the aberrant overexpression and excessive secretion of inflammatory cytokines via the MyD88-dependent pathway. Ultimately, this process disrupts the microenvironment essential for neural regeneration. In addition, Huang et al. (2021b) observed a reduction in the M2b macrophage subset accompanied by an expansion of the M1 macrophage population in patients with cognitive impairment. Activated M1 macrophages secrete proinflammatory mediators, including ROS, IL-1β, and TNF-α, fostering a microenvironment that impedes neural repair. Conversely, M2 macrophages, which exhibit an anti-inflammatory phenotype and promote tissue regeneration, contribute to neuroprotection; a decrease in this population exacerbates neurogenesis deficits. The core pathological mechanism of neuroinflammation is characterized by the pathological activation of microglia and astrocytes, along with the infiltration and migration of peripheral immune cells into the CNS. These changes trigger the cascading release of proinflammatory cytokines, chemokines, and ROS. The presence of these inflammatory mediators contributes to neuronal injury and impairs the regenerative capacity of damaged neurons, thereby promoting the development of PSCI.

### Oxidative stress exacerbates poststroke cognitive impairment

Emerging evidence indicates that oxidative stress plays a critical role in the pathogenesis of cognitive dysfunction. Upon cerebral ischemia or hypoxia, the function of the endogenous antioxidant defence system is markedly compromised, while ROS production is compensatorily enhanced. This imbalance between oxidative and antioxidant mechanisms disrupts neuronal homeostasis, thereby contributing to cognitive decline. Emerging evidence also highlights the crucial involvement of neurogenic processes in the regulation of cognitive function. In particular, aberrant hippocampal neurogenesis has been firmly established as a key factor involved in the pathogenesis and progression of diverse neurological disorders, including AD, depression, epilepsy, and schizophrenia (Wu and Zhang, 2023). The subgranular zone (SGZ) and subventricular zone (SVZ) of the dentate gyrus (DG) are among the most well-established niches for adult neurogenesis in the mammalian brain. A previous study has reported that stroke affects adult neurogenesis in the SVZ and DG (Wang et al., 2025). Zhang et al. (2024b) demonstrated that intracerebral hemorrhage (ICH) impairs hippocampal NSC proliferation and differentiation via the iron-ROS-Itga3 signaling axis. While this mechanism induces aberrant NSC activation in the early disease stages, it ultimately causes the depletion of the NSC pool and suppresses neurogenesis during the chronic phase, ultimately contributing to long-term cognitive impairment. As an antioxidant enzyme, superoxide dismutase (SOD)plays central roles in maintaining redox homeostasis and modulating inflammatory responses by catalyzing the disproportionation of superoxide anions into hydrogen peroxide and oxygen. Zhang et al. (2022b) showed that decreased SOD activity serves as a significant predictor of secondary cognitive impairment in patients with mild acute IS. Several studies have indicated that reduced SOD activity in both plasma and brain tissue is strongly associated with cognitive impairment in patients with various neurological disorders, including schizophrenia, cerebral small vessel disease (CSVD), and AD. In transgenic mouse models of AD, SOD deficiency has been shown to promote Aβ oligomerization, accelerate plaque formation, and exacerbate tau hyperphosphorylation. This impairment in SOD activity further contributes to the accumulation of ROS. In patients with subarachnoid hemorrhage, Yue et al. (2023) observed that hemoglobin-induced ROS activation triggers pyroptosis in NSC via the NLRP3/gasdermin D (GSDMD) pathway, thereby suppressing neurogenesis.

### Blood–brain barrier disruption promotes poststroke cognitive impairment

The BBB is a highly selective physiological barrier in the CNS. Histologically, it is composed of windowless ECs, pericytes, astrocyte processes, perivascular macrophages, the endothelium, and the basement membrane. Anatomically, the BBB constitutes a key component of the neurovascular unit (NVU). Its core structure consists of specialized ECs that interact closely with pericytes and astrocyte processes via the basement membrane. Under pathological conditions such as ischemic injury and cerebral hemorrhage, cellular components within the NVU can be activated, leading to a severe disruption of BBB integrity. This disruption allows aberrant infiltration of peripheral immune cells into the CNS and impairs the tightly regulated transport of molecules and ions. These processes induce vasogenic edema and secondary hemorrhage, thereby disrupting cerebral homeostasis and aggravating neuronal injury (Candelario-Jalil et al., 2022). Following stroke, the expression of matrix metalloproteinases (MMPs) is significantly upregulated. Elevated levels of MMPs directly impair BBB integrity through the proteolytic degradation of tight junction proteins. Liu et al. (2024a) found that hemorrhagic stroke triggers inflammatory reactive astrocytes, which contribute to BBB disruption through MMP-3. Alkhalifa et al. (2023) detected altered BBB permeability in patients with early and mild AD using gadolinium-based dynamic contrast-enhanced magnetic resonance imaging (DCE-MRI). Specifically, the soluble Aβ protein complex and lipoprotein receptor-related protein 1 (LRP-1) in the blood can be translocated into the cerebrospinal fluid (CSF) and brain parenchyma through the compromised BBB. Concurrently, an impaired BBB exhibits a reduced ability to clear Aβ, leading to the accumulation of neurotoxic Aβ oligomers within the brain interstitial fluid. Goulay et al. (2020) demonstrated that abnormal Aβ deposition in brain tissue contributes to synaptic impairment, leading to cognitive decline and the onset of dementia. BBB disruption and its associated pathological changes are likely to play critical roles in the development of PSCI.

### Neurotransmitter dysregulation leads to poststroke cognitive impairment development

Stroke disrupts the homeostasis of the neurotransmitter system, thereby contributing to cognitive impairment. The evidence suggests that chronic cerebral ischemia significantly reduces the levels of key neurotransmitters, including acetylcholine (ACh), dopamine (DA), gamma-aminobutyric acid (GABA), and glutamate. The dynamic equilibrium between the excitatory glutamatergic system and the inhibitory GABAergic system is essential for maintaining cognitive function and synaptic plasticity. Following cerebral ischemic injury, glutamate levels in the hippocampus decrease as a result of increased uptake through excitatory amino acid transporter 2. This increased neuronal glutamate transport elevates excitotoxic stress, contributing to neuronal damage and cognitive impairment (Verma et al., 2022). Chronic cerebral ischemia has been shown to reduce both the mRNA and protein levels of GABA and its receptor subtypes (GABA_A_ and GABA_B_) in the hippocampus, indicating impaired GABAergic neurotransmission. Learning and memory functions are significantly impaired by enhanced GABAergic activity within the hippocampus. Suppressing GABAergic synapse-induced neuronal hyperactivity can improve these cognitive functions. Notably, the excessive activation of GABAergic neurotransmission in the hippocampus following stroke suppresses long-term potentiation, which may represent a key mechanism underlying the development of PSCI. Research has indicated that reduced hippocampal levels of brain-derived neurotrophic factor (BDNF), a key regulator of learning and memory, following middle cerebral artery occlusion (MCAO) can lead to synaptic dysfunction in the ipsilateral hippocampus. Okamura et al. (2020) showed that the administration of the GABAA receptor antagonist bicuculline 2 weeks after stroke can significantly regulate the expression levels of neuroplasticity-related genes such as BDNF and synaptophysin in the affected hippocampus. These findings suggest that the overactivation of the GABAergic neurotransmitter system in the hippocampus after stroke may be involved in the pathogenesis of PSCI by inhibiting BDNF expression. Torres-López et al. (2023) observed that elevated GABA levels in the hippocampus following stroke in a mouse model of MCAO resulted in aberrant neurogenesis within the SGZ, thereby contributing to the development of PSCI.

**Figure 2:**
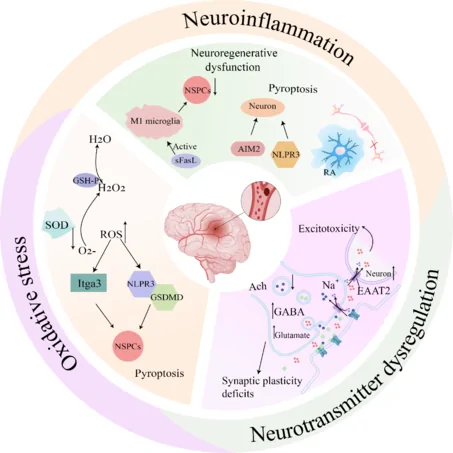
Mechanisms of poststroke cognitive impairment: Neuroinflammation, oxidative stress, and neurotransmitter imbalance. This illustration delineates three pivotal and independent pathological mechanisms underlying post-stroke cognitive impairment. (Top) Neuroinflammation: Following stroke, M1 microglia and reactive astrocytes contribute to neural stem/progenitor cells death via activation of the AIM2 inflammasome. (Left) Oxidative Stress: Increased production of reactive oxygen species after stroke induces neural stem/progenitor cells death by activating Itga3 and NLRP3. (Right) Neurotransmitter Dysregulation: Deficits in synaptic plasticity lead to dysregulation of neurotransmitters, directly contributing to the onset of PSCI. Ach: Acetylcholine; AIM2: absent in melanoma 2; EAAT2: excitatory amino acid transporter 2; GABA: γ-aminobutyric acid; MDA: malondialdehyde; NSPCs: neural stem/progenitor cells; RA: reactive astrocytes; ROS: reactive oxygen species; SOD: superoxide dismutase.

In the cerebral cortex, ACh plays an extensive role in regulating diverse cortical functions through its excitatory neuromodulatory effects. ACh is primarily localized to the axons of cholinergic neurons and is synthesized in a single-step reaction catalyzed by choline acetyltransferase (ChAT), which combines choline and acetyl-CoA. The transport and storage of ACh into synaptic vesicles are mediated by the vesicular acetylcholine transporter. As a key neurotransmitter involved in learning and memory, the levels of ACh are often markedly reduced following stroke, thereby contributing to cognitive impairment. Kim et al. (2020b) demonstrated that 4 weeks after bilateral common carotid artery occlusion, hippocampal ChAT expression is upregulated, whereas acetylcholinesterase (AChE) activity is significantly downregulated. This imbalance leads to decreased ACh levels, ultimately impairing synaptic plasticity (**Figure 2**).

### White matter lesions increase the risk of poststroke cognitive impairment

White matter lesions (WMLs) appear as periventricular hypodense areas on CT scans, along with prominent high-signal intensities on T2-weighted MRI images. While the traditional view posits that gray matter is the primary substrate for cognitive functions, accumulating evidence underscores the significant contribution of white matter to cognitive processes. Guo and Shi (2022) confirmed that ventricular enlargement and an increased white matter hyperintensity (WMH) volume significantly correlate with cognitive impairment. Kort et al. (2023) demonstrated that, compared with controls, stroke survivors display more severe white matter neurodegeneration in bilateral regions, including the thalamus, striatum, and cerebellar tracts. Liu et al. (2025a) reported that cerebral white matter injury can induce high signal intensity in periventricular white matter regions and affect structural integrity in areas such as the bilateral corpus callosum, fornix, hippocampal cingulum, and anterior connectivity pathways. Furthermore, Hu et al. (2022) observed that patients with WMLs exhibit reduced fractional anisotropy, along with increased mean diffusivity, axial diffusivity, and radial diffusivity. These microstructural alterations are associated with cognitive decline. Ball et al. (2022) showed that WMLs are associated with a threefold increased risk of PSCI. Additionally, Coenen et al. (2024) reported that the WMH volume in the forceps major correlates with slower processing speed in patients. Notably, the WMH volume in the left anterior thalamic radiation was associated with impairments in attention, executive function, and information processing speed. Schellekens et al. (2025) documented alterations in the white matter microstructure among patients with subacute stroke. Together, these findings substantiate that poststroke WMLs significantly contribute to the development of PSCI.

### Dysfunctional myelin repair and axonal regeneration cause poststroke cognitive impairment

**Figure 3:**
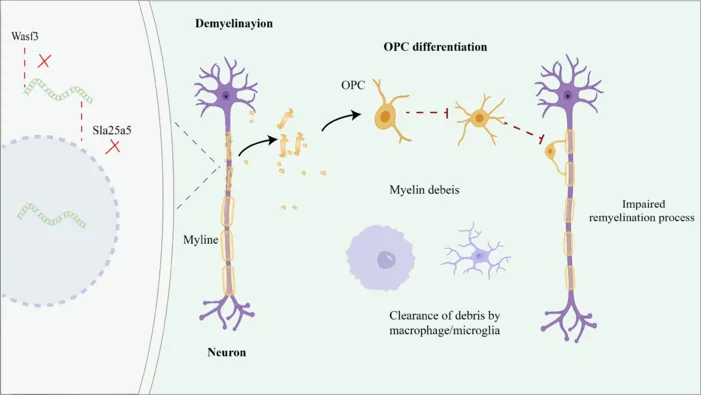
The impaired remyelination following stroke contributes to the development of PSCI. This figure illustrates the core pathological processes of demyelination and impaired remyelination in the central nervous system following PSCI. The left panel demonstrates that impaired differentiation of oligodendrocyte precursor cells is a key factor in remyelination failure. The right panel indicates that abnormalities in the *Sla25a5* and *Wasf3* genes can lead to myelin damage. OPC: Oligodendrocyte precursor cell; PSCI: poststroke cognitive impairment.

Oligodendrocytes are responsible for producing the myelin sheath, which enables faster signal transmission and provides essential metabolic support to neurons. Studies have shown that myelin plasticity underlies higher cognitive functions such as learning and memory. Using MRI, Khodanovich et al. (2021) successfully tracked the progression of striatal myelin injury and repair from the acute to chronic phases following MCAO, providing a crucial methodological framework for investigating poststroke myelin pathology. Qian et al. (2025) identified key molecular mechanisms underlying poststroke myelin damage, revealing that dysfunctional Wasf3 and Slc25a5 genes are primary contributors to myelin loss. Additionally, Mercier et al. (2024) demonstrated that, although the cerebral cortex is capable of remyelination following demyelination, the newly formed myelin fails to maintain normal function. This aberrant remyelination alters neural network properties and contributes to cognitive deficits. Thus, impaired myelin regeneration may represent a critical pathological mechanism in the development of PSCI (**Figure 3**).

Axonal regeneration is an essential physiological process for self-repair and functional recovery in the brain following a stroke. Ueno et al. (2023) observed substantial axonal and dendritic shedding during the acute phase of stroke, followed by progressive regeneration of affected axons in the chronic recovery phase. Furthermore, in a MCAO mouse model, the expression of the axonal marker phosphorylated neurofilament heavy chain (pNfH) was significantly decreased on day 7 but returned to baseline levels by Days 28 and 56, indicating an inherent self-repair capacity within the CNS after stroke.

Recent studies have indicated that L-carnitine has multiple therapeutic effects, including neuroprotective properties and the promotion of nerve fiber regeneration. Research in rat models of cerebral ischemia has revealed its potent antioxidant capacity, which contributes to reduced neuronal injury (Guerreiro et al., 2025). In a study using a model of cerebral hypoperfusion, the administration of L-carnitine (600 mg/kg/d) for 28 consecutive days improved the memory capacity of mice. However, clinical observations have shown a significant reduction in blood L-carnitine levels among stroke patients. This metabolic deficiency may impair axonal growth and myelin repair, thereby contributing to the development of cognitive dysfunction.

### Synaptic dysfunction exacerbates poststroke cognitive impairment

Synaptic plasticity refers to the ability of synapses to be formed and eliminated, as well as to modify their electrophysiological, molecular, and structural properties. It serves as a fundamental mechanism regulating cognitive function (Li et al., 2024a). Studies have found that stroke can severely impair synaptic plasticity, which may represent an important pathophysiological basis for PSCI (Hong et al., 2021; Chi et al., 2023). Moreover, the homeostatic regulation of synaptic function relies on a complex signaling network in which inflammation-related molecules play a pivotal role in modulating synaptic plasticity. In individuals with pathological conditions such as stroke, the aberrant production of pro-inflammatory factors activates the NF-κB signaling pathway, initiating an inflammatory cascade. Additionally, neuroinflammatory responses impair synaptic plasticity in regions such as the cerebral cortex, striatum, and hippocampus by activating microglia and astrocytes (Baraibar et al., 2024). Furthermore, excess TNF-α has been shown to impair synaptic plasticity in cortical pyramidal neurons by disrupting intracellular calcium homeostasis and interfering with signaling pathways mediated by synaptic surface proteins (Kleidonas et al., 2023). A growing number of studies have revealed important roles for neurotrophic factors such as BDNF and nerve growth factor n the regulation of synaptic plasticity (Wu et al., 2024). Wang et al. (2022a) revealed that BDNF plays crucial roles in maintaining neuronal morphology, differentiation, and regeneration after injury, as well as in promoting axon growth and restoring synaptic function through its specific receptor, tropomyosin receptor kinase B. Notably, the study by Wang et al. (2024b) indicates that BDNF levels are significantly reduced in patients with PSCI compared to healthy controls. Moreover, nerve growth factor modulates the expression of cholinergic neuronal markers through the p75 and TrkA receptors, profoundly influencing structural synaptic plasticity by promoting long-term potentiation, stimulating neurite outgrowth, and enhancing synaptic plasticity (Jiao et al., 2023). In addition, Wu et al. (2024a, b) demonstrated that the significant downregulation of the SIRT1/BDNF signaling pathway in hippocampal CA1 region glutamatergic neurons in aged mice triggers impaired synaptic plasticity, ultimately leading to PSCI. Furthermore, Li et al. (2025) reported that curcumin significantly enhances synaptic plasticity and cognitive function in mice with hypoxic–ischemic brain injury by upregulating the expression of PSD95 and BDNF. These findings strongly support the view that impaired synaptic plasticity represents a critical mechanism underlying PSCI and highlight potential targets for clinical intervention.

## Biomarkers of Poststroke Cognitive Impairment

### Metabolomic biomarkers

#### Plasma neurofilament light chain, plasma neurofilament heavy chain, brain-derived neurotrophic factor, vascular endothelial growth factor, and neuropeptide Y

Neurofilaments (Nfs) are key neuronal proteins synthesized in the neuronal soma and assembled into intermediate filaments within axons. Nfs are categorized into three major subtypes: neurofilament light chain (NfL), medium chain (NfM), and heavy chain (NfH). Among these proteins, NfL is a neuron-specific structural protein whose aberrant expression is significantly positively correlated with the degree of axonal damage and the progression of neurodegenerative diseases. A systematic meta-analysis confirmed that elevated plasma NfL levels serve as a reliable biomarker for predicting the functional prognosis of patients with IS. Wang et al. (2021) reported that elevated plasma NfL (pNfL) levels measured within 48 hours after acute anterior circulation IS onset predict the risk of PSCI at 90 days. Similarly, Jiang et al. (2022) showed that early pNfL levels (within 48 hours of onset) are independently associated with the 90-day risk of cognitive impairment in patients with posterior circulation stroke. Research has demonstrated that neuronal damage following stroke can lead to the release of neuronal proteins into the CSF and blood. One study showed that the level of phosphorylated NfH (pNfH) chain protein is upregulated in individuals with various CNS disorders, including acute IS. Notably, the latest findings reported by Qiao et al. (2025) further confirm that elevated pNfH levels are present not only in patients with PSCI but also in those who experience cognitive decline 1 year after stroke. Multivariate regression analysis indicated that after adjusting for confounding factors such as age and education level, pNfH levels remained an independent risk factor for PSCI, providing a new molecular target for the early prediction and intervention of PSCI.

As a core member of the neurotrophin family, BDNF has multiple critical functions in the CNS. Amidfar et al. (2024) reported that BDNF plays a decisive role in regulating neuronal survival, maturation, and synapse formation; mediating synaptic plasticity and neurogenesis; and maintaining and enhancing cognitive functions such as learning and memory. BDNF is predominantly enriched in the CNS but is also detectable in the peripheral circulation, making it a promising diagnostic biomarker for neurological disorders. Additionally, serum BDNF levels are significantly decreased in patients with dementia, and the extent of this reduction is strongly positively correlated with the severity of cognitive impairment (Nikolac Perkovic et al., 2023). Chang et al. (2024) found that among IS patients, those in the highest serum BDNF tertile had a 40% lower risk of PSCI at 3 months than those in the lowest tertile. Chen et al. (2018) documented markedly reduced serum BDNF levels in stroke patients, with the concentrations positively correlated with Mini-Mental State Examination scores. Havlovska et al. (2023) further confirmed that serum BDNF levels are significantly reduced during the acute phase of IS and that low BDNF levels, combined with the thrombotic stroke subtype, serve as an independent risk factor for acute cognitive impairment. Similarly, Hassan and Yarube (2018) observed that decreased peripheral BDNF levels were significantly associated with a higher incidence of PSCI over a 10-month follow-up period. Vascular endothelial growth factor (VEGF) is a dimeric glycoprotein that plays multiple roles in angiogenesis and neuroprotection. Prodjohardjono et al. (2020) reported that stroke patients with VEGF concentrations ≥ 519.8 pg/mL have a significantly higher risk of developing PSCI at 3 months. Furthermore, combining VEGF levels with infarct volumes ≥ 0.054 mL improved the accuracy of PSCI prediction. Neuropeptide Y is a widely expressed 36-amino acid neurotransmitter that is notably abundant in the mammalian nervous system. It regulates diverse functions, including mood, appetite, circadian rhythms, neuroplasticity, and neurotrophic support. Dong et al. (2022) found that elevated plasma neuropeptide Y levels remained significantly associated with a reduced risk of PSCI, even after adjusting for confounding factors.

#### Homocysteine

Homocysteine (Hcy) is a nonproteinogenic amino acid containing a thiol group that is metabolized into cysteine analogues via the methionine pathway. Wu et al. (2020) observed elevated Hcy levels in patients with PSCI suggesting that Hcy may serve as an independent risk factor for PSCI. Zhu et al. (2023a) showed that patients with thalamic infarction and cognitive impairment, assessed using the Montreal Cognitive Assessment, exhibited significantly higher Hcy levels than those with normal cognitive function. The combination of Hcy and non-high-density lipoprotein cholesterol (non-HDL-C) levels had a significant predictive value for PSCI, with an area under the curve (AUC) of 0.769. Additionally, one study indicated that Hcy levels are an associated risk factor for cognitive impairment in patients with progressive IS. The combined detection of Hcy and the S100B protein demonstrated strong predictive efficacy for cognitive dysfunction following progressive IS, with a combined AUC of 0.739 (Li et al., 2023). Li et al. (2021) found that in female patients with mild acute IS or transient ischemic attack, Hcy levels were significantly associated with the 12-month risk of PSCI. Their findings demonstrate that estrogen exerts a protective effect on the vascular endothelium. Notably, postmenopausal women exhibit a dual pattern of significant changes: decreasing estrogen levels and increasing Hcy levels. These changes may increase endothelial impairment and accelerate the progression of PSCI. Accumulating evidence indicates that elevated Hcy levels may contribute to the pathogenesis of PSCI by inducing oxidative stress, promoting vascular inflammation, impairing endothelial function, and accelerating the pathological deposition of Aβ and tau proteins. Furthermore, increased plasma Hcy levels lead to hyperhomocysteinemia, which has long been recognized as an important risk factor for systemic atherosclerosis and cardiovascular disease. Elevated hyperhomocysteinemia levels are also associated with various neurological disorders, particularly cognitive impairment and stroke.

#### Cystatin C

As a key member of the endogenous cysteine protease inhibitor family, cystatin C (CysC) is constitutively secreted by nucleated cells and is detectable at measurable concentrations in various biological fluids. Accumulating evidence indicates complex associations between CysC levels and cognitive outcomes following stroke. Guo et al. (2020) reported elevated CysC levels in patients with acute IS and normal renal function that were significantly associated with a reduced risk of cognitive impairment at the 3-month follow-up. However, Zeng et al. (2019) found that elevated CysC levels and the presence of the CST3 B allele significantly increase the risk of VCI in patients with acute cerebral infarction. Yan et al. (2022a) reported that patients with mild IS presenting with PSCI had significantly higher baseline CysC levels. An elevated CysC level was found to independently predict the presence of PSCI at 3 months, with a sensitivity of 79.9% and a specificity of 58.1%. Zuo et al. (2023) reported a significant U-shaped relationship between serum CysC levels and overall cognitive function at 1 year in patients with acute IS or TIA.

#### Serum uric acid levels and the serum uric acid/serum creatinine ratio

Serum uric acid (SUA), the final metabolite of purine nucleotide metabolism, has notable antioxidant properties in humans. It functions by scavenging oxygen free radicals and inhibiting lipid peroxidation. Conversely, elevated SUA levels in patients with vascular dementia are associated with cerebral white matter atrophy and may contribute to worsened cognitive impairment. Ran et al. (2020) identified a significant association between elevated SUA levels (> 406 μM) and better cognitive function in patients with cerebral infarction. However, SUA levels were significantly higher in patients with PSCI than in those with normal cognitive function. Furthermore, a logistic regression analysis confirmed that SUA was an independent risk factor for PSCI. Xu et al. (2023b) demonstrated that in patients with acute mild IS, cognitive impairment could be effectively predicted when SUA levels exceeded 363.58 μmol/L. Subgroup analyses further indicated that the association between SUA levels and the risk of PSCI remained significant even after adjusting for potential confounders. Yan et al. (2022b) confirmed that patients with acute IS presenting with higher SUA levels had a greater likelihood of developing PSCI than those with lower levels. Additionally, Liu et al. (2021) reported a U-shaped relationship between SUA and PSCI in male patients, where both low and high SUA levels were associated with an increased incidence of cognitive impairment.

The SUA-to-serum creatinine ratio (SUA/SCr) has been extensively studied in various diseases. Songsomboon et al. (2020) found that the degree of reduction in the SUA/SCr in patients with Parkinson’s disease (PD) was significantly negatively correlated with the clinical stage of the disease. More importantly, in young people with acute stroke, the SUA/SCr is significantly positively correlated with the risk of stroke recurrence, and a lower SUA/SCr is associated with poor functional outcomes in patients with acute IS (Xu et al., 2024a). Additionally, a lower SUA/SCr is associated with an increased risk of cognitive impairment in Americans aged 60 years and older (Chen et al., 2024). Similarly, Zhou et al. (2025) suggested that the SUA/SCr may be a potential indicator of cognitive decline in middle-aged adults. Furthermore, Liao et al. (2025) reported that the SUA/SCr at admission was an independent predictor of early PSCI and a protective factor against early PSCI, indicating that individuals with lower SUA/SCr values had an increased risk of PSCI.

#### Polyunsaturated fatty acid, L-carnitine, trimethylamine-N-oxide, and choline

Dietary factors are significantly associated with cognitive impairment. Kotlęga et al. (2021a) reported a strong association between eicosanoid levels and cognitive function in stroke patients. Metabolically, α-linolenic acid acts as a precursor for γ-linolenic acid, which is further converted into arachidonic acid via enzymatic processes. In a subsequent study, Kotlęga et al. (2021b) demonstrated that disturbances in polyunsaturated fatty acid metabolism may contribute to the development of PSCI. Interestingly, Szczuko et al. (2020) suggested that within the pathophysiology of IS, arachidonic acid may serve as a key mediator that regulates the interplay among lipid metabolism, neuroinflammatory responses, and cognitive dysfunction. L-Carnitine, the biologically active isomer of carnitine, is classified as a micronutrient primarily obtained from animal-based foods, particularly red meat and dairy products. Che et al. (2022) reported that higher plasma L-carnitine levels were negatively associated with cognitive dysfunction at 3 months after IS. Furthermore, stroke patients with elevated serum L-carnitine concentrations and reduced levels of systemic inflammatory markers exhibited a significantly lower risk of PSCI. Dietary L-carnitine is metabolized by the gut microbiota to produce trimethylamine-N-oxide (TMAO). In fact, several dietary nutrients, including choline, phosphatidylcholine, and L-carnitine, are processed by gut microbes to generate TMAO. Current evidence indicates that TMAO plays a significant role in vascular pathologies. Moreover, elevated TMAO levels inhibit mechanistic target of rapamycin signaling, resulting in synaptic damage and decreased expression of plasticity-related proteins, ultimately contributing to brain ageing and cognitive impairment. Zhu et al. (2020) reported that elevated plasma TMAO levels in IS patients were associated with cognitive impairment at 1 year, suggesting its potential as an independent predictor of PSCI. Similarly, Wang et al. (2023b) demonstrated that TMAO may contribute to cognitive decline 3 months after a transient ischemic attack (TIA) through the upregulation of inflammatory markers, including C-reactive protein (CRP). Furthermore, Gong et al. (2021) indicated that higher plasma TMAO concentrations measured upon hospital admission could serve as a potential biomarker for predicting PSCI. Choline is an essential nutrient that plays a vital role in the human body by supporting the synthesis of the neurotransmitter acetylcholine, maintaining cell membrane integrity, and facilitating methyl group metabolism. Zhong et al. (2021) further showed that in patients with IS, higher levels of choline and betaine are associated with a reduced risk of cognitive impairment.

#### Glycosylated hemoglobin, hemoglobin, hemoglobin-to-red cell distribution width ratio, and prognostic nutritional index

Glycosylated hemoglobin (HbA1c), which reflects the average blood glucose level over the past 3 months, is widely used in clinical practice to assess glycemic control. Xu et al. (2023a) identified a nonlinear relationship between HbA1c levels and the PSCI incidence. Specifically, diabetic patients with HbA1c levels exceeding 8.2% had a higher incidence of PSCI than those in the control group. Hemoglobin (Hb), the primary component of red blood cells, binds oxygen to form oxyhemoglobin and facilitates its delivery throughout the body. Li et al. (2015) reported that approximately 40% of patients with acute IS exhibit decreased Hb levels upon hospital admission. Xu et al. (2023d) further demonstrated that lower Hb levels are significantly associated with an increased incidence of PSCI. Jia et al. (2022) showed that patients with acute IS or TIA who presented with higher Hb levels within three months after onset had a reduced risk of PSCI.

The hemoglobin-to-red cell distribution width ratio is an emerging biomarker calculated by dividing the Hb level by the red cell distribution width. Liu et al. (2025b) found that a lower baseline hemoglobin-to-red cell distribution width ratio was associated with cognitive impairment one month after stroke. Optimal nutrition is essential for maintaining and improving cognitive function. Current research has shown the value of the prognostic nutritional index as a comprehensive assessment tool for evaluating the nutritional status of patients and their prognosis. Wang et al. (2024b) reported that a lower prognostic nutritional index was an independent predictor of PSCI and was significantly associated with overall cognitive function and attention domains. As a potential biomarker, this index can be used to effectively screen high-risk individuals with an abnormal immune nutritional status and provides an easy method for the early identification of PSCI.

#### High-density lipoprotein-C, triglycerides/high-density lipoprotein-C, low-density lipoprotein, and non-high-density lipoprotein-C/high-density lipoprotein-C

Lipoproteins are biomolecular complexes composed of lipids such as triglycerides (TG) and cholesterol esters bound to apolipoproteins via noncovalent interactions. They are classified into five major categories based on a density gradient: high-density lipoprotein (HDL), low-density lipoprotein (LDL), intermediate-density lipoprotein (IDL), very low-density lipoprotein (VLDL), and chylomicrons. Wang et al. (2024a) suggested that in patients with acute IS, lower baseline HDL cholesterol (HDL-C) levels were associated with better cognitive function at 6 months in those with MCI. However, this correlation was observed only within specific concentration ranges: at low concentrations (< 0.92 mmol/L) and in the medium-to-high range (1.24–1.70 mmol/L). Additionally, the TG/HDL-C ratio, a sensitive indicator of dyslipidemia, was initially established as significantly and positively correlated with the homeostatic model assessment of insulin resistance. In a related study, Cheng et al. (2022) demonstrated that higher baseline TG/HDL-C ratios in patients with acute IS were significantly and positively correlated with the risk of PSCI at 3 months. A previous study has shown that LDL cholesterol (LDL-C) levels can be used to respond to the effectiveness of lipid therapy. Wu et al. (2020) found that LDL is a risk factor for PSCI. An elevated nonhigh-density lipoprotein cholesterol (non-HDL-C) level was first associated with cognitive impairment in 2013, when significantly higher levels were observed in patients with MCI than in controls. Wang et al. (2022e) found that increased serum non-HDL-C levels may increase the risk of cognitive impairment in patients with ICH. Similarly, Shi et al. (2024) reported that patients who developed cognitive impairment after cerebral infarction had a significantly higher non-HDL-C/HDL-C ratio than those with normal cognitive function, indicating that this ratio may serve as a potential risk factor for PSCI.

#### Thiamine and retinoic acid

Thiamine is a form of vitamin B1, and thiamine deficiency or functional thiamine insufficiency are characteristics of AD. Feng et al. (2020) identified thiamine deficiency as an independent predictor of early cognitive impairment in patients with cerebral infarction. Serum levels of folate and its metabolic marker, methylmalonic acid, have been proposed as potential predictive biomarkers for PSCI. Retinoic acid (RA), the active form of vitamin A derived from retinol metabolism, exhibits unique vascular endothelial protective properties among retinoids. Animal experiments have confirmed that treatment with RA significantly reduces ischemic brain damage and improves neurological function. Hou et al. (2019) found that after correcting for age, education, and vascular risk factors, baseline serum RA levels were associated with an increased incidence of PSCI at 3 months in patients with IS.

#### Alkaline phosphatase and cortisol

Alkaline phosphatase (ALP) is a zinc-dependent hydrolase expressed in organisms ranging from prokaryotic microorganisms to eukaryotes. Jia et al. (2020) reported that patients in the PSCI group had significantly higher serum ALP levels than those in the control group. Cortisol, the primary glucocorticoid in the human body, is synthesized and secreted through processes regulated by the hypothalamic–pituitary–adrenal (HPA) axis. Unlike serum measurements, salivary cortisol levels are unaffected by variations in the corticosteroid-binding globulin concentration and are highly consistent across individuals with different demographic characteristics, including age, sex, body mass index, and smoking status. Current evidence indicates that elevated salivary cortisol levels due to HPA axis dysfunction may contribute to cognitive impairment. Wang et al. (2021b) further demonstrated that salivary cortisol exhibits a strong diagnostic performance for mild PSCI, with a sensitivity of 0.973 and a specificity of 0.980.

#### Osteoprotegerin

Osteoprotegerin (OPG) is a key protein that regulates bone metabolism, primarily by inhibiting osteoclast formation to maintain bone strength. A clinical study has shown that elevated plasma OPG concentrations can predict poor outcomes in stroke patients, including functional disability, death, and the occurrence of vascular events (Zhu et al., 2023b). Notably, another study revealed that OPG levels are associated with cognitive impairment and the occurrence of emotional disorders (Duggan et al., 2023). Chang et al. (2025) showed that elevated plasma OPG levels are associated with an increased risk of PSCI within 3 months.

#### Dipeptidyl peptidase-4

Dipeptidyl peptidase-4 (DPP4) is a transmembrane serine exopeptidase that cleaves N-terminal dipeptides via its extracellular catalytic domain. It can also be hydrolyzed and released into the circulation as a soluble, enzymatically active form (sDPP4). DPP4 is known to inactivate intestinal hormones, thereby influencing glucose metabolism, cell migration, differentiation, and signal transduction. Although DPP4 inhibitors reduce DPP4 enzymatic activity, they paradoxically increase circulating sDPP4 levels. These inhibitors are considered promising agents for dementia prevention, as they may reduce Aβ accumulation and tau phosphorylation (Biessels et al., 2019). A previous study has also indicated their potential to reverse memory impairment (Gault et al., 2015). You et al. (2023) were the first to show that higher circulating sDPP4 levels are associated with a reduced risk of PSCI in patients with noncardioembolic acute IS.

#### Adiponectin

Adiponectin (APN), an adipocyte-specific secreted protein, is involved in diverse physiological and pathological functions, such as anti-inflammatory and anti-atherosclerotic effects, as well as the regulation of insulin sensitivity (Li et al., 2024a, b). Within the CNS, APN has been shown to participate in multiple physiological and pathological processes, including the regulation of cognitive function (Kim et al., 2022). Li et al. (2022b) reported significantly lower serum APN levels in patients with VCI than in cognitively normal controls. Furthermore, Quan et al. (2022) demonstrated that serum APN levels were negatively correlated with the severity of WMLs and positively correlated with Montreal Cognitive Assessment scores. These findings suggest that APN may act as a protective factor against cognitive impairment (**Additional Table 1**).

**Additional Table 1:** Summary of metabolic biomarkers of poststroke cognitive impairment

Reference	Adjust age and gender	Assessment of poststroke cognitive Impairment	Biomarker	Strengths	Limitations	Significance
Wang et al., 2021d	Yes	Montreal Cognitive Assessment	Plasma neurofilament light chain	Large sample size; Standardized biomarker measurement using SiMoA platform	Single-center design; Exclusion of severe cases; Single time-point biomarker measurement	Predicts 3 months poststroke cognitive impairment
Chang et al., 2024	Yes	Mini-Mental State Examination Montreal Cognitive Assessment	Brain-derived neurotrophic factor	Large sample size; First multicenter study	No baseline cognitive assessment; Relatively young sample	Predicts 3 months poststroke cognitive impairment
Prodjohardjono et al., 2020	Yes	Montreal Cognitive Assessment National Institutes of Health Stroke Scale	Vascular endothelial growth factor	First study to link acute serum vascular endothelial growth factor levels with poststroke cognitive impairment risk	Small sample size; Measured only once, missing dynamic changes	Predicts 3 months poststroke cognitive impairment
Dong et al., 2022	Yes	National Institutes of Health Stroke Scale Montreal Cognitive Assessment	Neuropeptide Y	Large sample size; Measured using standardized ELISA	Measured only once, missing dynamic changes; Excluded severe cases	Predicts 3 months poststroke cognitive impairment
Li et al., 2021	No	Montreal Cognitive Assessment National Institutes of Health Stroke Scale	Homocysteine	Large prospective cohort; Measured using highly sensitive SiMoA technology	Single-center design; excluded severe cases; Measured only once, missing dynamic changes;	Predicts 3 months and 12 months poststroke cognitive impairment
Yan et al., 2022a	Yes	Montreal Cognitive Assessment	Cystatin C	First study focusing on Cystatin C and poststroke cognitive impairment	Single measurement; Moderate sample size with some loss to follow-up.	Predicts 3 months poststroke cognitive impairment
Liu et al., 2021	Yes	Montreal Cognitive Assessment	Serum uric acid	Large sample size	Measured only once at baseline;	Predicts 3 months poststroke cognitive impairment
Che et al., 2022	Yes	Montreal Cognitive Assessment National Institutes of Health Stroke Scale	L-carnitine	Used advanced mass spectrometry for precise L-carnitine measurement	Single baseline measurement of L-carnitine; Excluded severe cases	Predicts 3 months poststroke cognitive impairment
Zhu et al., 2020	Yes	Mini-Mental State Examination	Trimethylamine-N-oxide	Prospective longitudinal design with 1-year follow-up	Single-center study with relatively small sample size;	Predicts1 year poststroke cognitive impairment
Zhong et al., 2021	-	Montreal Cognitive Assessment National Institutes of Health Stroke Scale Mini-Mental State Examination	Choline	Prospective design with a well-defined cohort from a multicenter trial	Excluded severe cases; Lack ofbaseline cognitive status data	Predicts 3 months poststroke cognitive impairment
Xu et al., 2023a	Yes	Montreal Cognitive Assessment National Institutes of Health Stroke Scale	Glycated hemoglobin	Uses rigorous statistical methods (spline fitting)	Excluded severe cases; Small sample size;	Predicts 3-6 months poststroke cognitive impairment
Li et al., 2022c	Yes	Montreal Cognitive Assessment	Hemoglobin; Adiponectin	Uses well-defined clinical assessments	Small sample size; No longitudinal follow-up	Predicts 3 months poststroke cognitive impairment
You et al., 2023	Yes	Montreal Cognitive Assessment National Institutes of Health Stroke Scale	Soluble dipeptidyl peptidase-4	First large-scale study	Excluded severe cases; Measured only once, missing dynamic changes	Predicts 3 months poststroke cognitive impairment

ELISA: Enzyme-linked immunosorbent assay; SiMoA: single molecule array.

### Biomarkers of inflammation

#### C-reactive protein

CRP is a plasma protein synthesized by the liver and serves as a sensitive biomarker of systemic inflammation. Elevated CRP levels reflect an acute inflammatory response following ischemic cerebral infarction and are associated with a poor stroke prognosis and increased mortality. In a 10-year longitudinal study involving 5267 participants, high-sensitivity CRP levels were significantly elevated and positively associated with the incidence of cognitive impairment (Zheng and Xie, 2018). Irimie et al. (2018) further showed that elevated CRP levels are linked to cognitive impairment and that combining CRP with triiodothyronine (T3) levels predicts the likelihood of cognitive impairment at discharge with an accuracy of 80.42%. Similarly, Wang et al. (2023b) reported that among TIA patients, serum CRP concentrations were significantly higher in those with cognitive dysfunction than in those with normal cognitive function.

#### Interleukin-6

Interleukin-6 (IL-6) is a key cytokine that plays a central role in maintaining physiological homeostasis. Sandvig et al. (2023) identified a significant negative correlation between increased IL-6 levels and lower MoCA scores at 3, 18, and 36 months. Similarly, Wang et al. (2022d) found that higher IL-6 levels were independently associated with the development of cognitive impairment following IS or TIA. Loga-Andrijić et al. (2021b) observed that among female patients with acute IS, IL-6 levels were significantly increased in those with cognitive impairment compared with those with normal cognitive function. After the mean IL-6 concentration was adjusted for sex, a stratified analysis revealed a statistically significant difference in the IL-6 concentration in male patients (Loga-Andrijić et al., 2021a). One potential explanation for higher IL-6 levels in female patients with stroke experiencing cognitive impairment may involve hormonal influences.

#### Serum amyloid A

Serum amyloid A (SAA) is a low-molecular-weight protein comprising 104 amino acids that is primarily synthesized in the liver. Cao and Chen (2020) reported a significant positive correlation between SAA levels and the severity of early cognitive dysfunction in an elderly population. Similarly, Zhang et al. (2021) observed higher SAA levels in patients who developed PSCI at 3 months. Ye et al. (2022) further showed that SAA levels were significantly increased in patients with PSCI compared with non-PSCI patients. After multivariate adjustment, SAA showed a predictive efficacy for PSCI, with an AUC of 0.860.

#### Quinolinic acid/kynurenic acid

The inflammatory response following stroke, particularly the activation of macrophages by Th1 cells via the IFN-γ signaling pathway, may contribute to the development of PSCI. IFN-γ stimulates macrophages to secrete neopterin and activates the kynurenine pathway. Furthermore, IFN-γ is significantly associated with cognitive dysfunction through specific activation of the indoleamine 2,3-dioxygenase signalling pathway and modulation of its enzymatic activity (Cogo et al., 2021). The indoleamine 2,3-dioxygenase-mediated enhancement of the kynurenine pathway leads to significant increases in the levels of kynurenic acid (KA) and quinolinic acid (QA). These metabolites regulate synaptic plasticity through the modulation of glutamatergic synaptic transmission. The neuroexcitotoxicity of QA, a selective NMDA receptor agonist, is competitively inhibited by high concentrations of KA. The experimental evidence suggests that the QA/KA ratio may serve as an indicator of excitotoxicity mediated by NMDA receptor activation (Lautrup et al., 2019). Furthermore, Cogo et al. (2021) observed an elevated QA/KA ratio following stroke, which was associated with cognitive impairment at 3 months poststroke.

#### Neutrophil-to-lymphocyte ratio

The neutrophil-to-lymphocyte ratio (NLR), a sensitive marker of systemic inflammation, has gained recognition in recent years for its prognostic value across various pathological conditions, including cardiovascular disease and chronic inflammatory disorders (Zhang et al., 2024a). Halazum et al. (2014) reported higher NLR in patients who developed cognitive impairment following carotid endarterectomy. A Korean study documented that an elevated NLR during the acute phase of stroke is associated with cognitive deficits, particularly in memory and visuospatial function, at 3 months poststroke (Lee et al., 2021). NLR were significantly higher in patients with PSCI than in those with normal cognitive function, and this difference remained significant after adjustment for confounders such as age (Zha et al., 2021). Shang et al. (2022) investigated the value of the peripheral blood neutrophil percentage and NLR for predicting PSCI in patients with acute IS. Their results indicate that an increased neutrophil percentage and NLR are associated with an increased risk of PSCI, suggesting that these biomarkers may have diagnostic utility in predicting cognitive impairment after stroke.

#### Systemic immune–inflammatory index, systemic inflammatory response index, hemoglobin, albumin, lymphocyte, and platelet score, and lactate dehydrogenase-to albumin ratio

The systemic immune–inflammatory index (SII) is a novel composite measure that integrates neutrophil, platelet, and lymphocyte counts to provide a more comprehensive reflection of the body’s immune and inflammatory status than individual inflammatory markers. A recent study has highlighted the predictive value of the SII for PSCI (Bao et al., 2023). Bao et al. (2023) demonstrated that a higher SII at 3 months is significantly associated with cognitive impairment in patients with IS, even after adjustment for potential confounders. Additionally, a novel systemic inflammatory response index (SIRI) has been proposed to assess systemic inflammation by integrating neutrophil, monocyte, and lymphocyte counts. Xia et al. (2023) reported that elevated SIRI is associated with an increased risk of stroke. Wang et al. (2022c) found that older adults with higher SIRI scores were more likely to exhibit cognitive impairment. Similarly, a study focusing on MCI in older adult individuals reached consistent conclusions (Liu et al., 2023). Chu et al. (2024) further demonstrated that a high SIRI at admission was independently associated with PSCI, yielding an AUC of 0.716, which underscores its significant predictive value for PSCI. The hemoglobin, albumin, lymphocyte, and platelet (HALP) score, a composite index derived from hemoglobin levels, albumin levels, lymphocyte counts, and platelet counts, serves as a novel marker for evaluating the systemic inflammatory status and nutritional levels. The HALP score is a reliable tool for assessing systemic inflammation and the nutritional status of patients with various malignancies and has prognostic value. Xu et al. (2023c) further confirmed that lower HALP scores are associated with an increased risk of early PSCI within 2 weeks after stroke. Additionally, the lactate dehydrogenase-to albumin ratio (LAR) has emerged as a new parameter for evaluating systemic inflammation and the nutritional status. Xu et al. (2022) reported that patients with PSCI exhibited significantly higher LAR levels than those without cognitive impairment.

#### High mobility group protein B1

HMGB1, a highly conserved nuclear protein, is involved primarily in maintaining genomic stability and regulating transcription under physiological conditions. Following stroke, HMGB1 is released by neurons and contributes to inflammatory processes. As a key regulatory factor, HMGB1 drives chronic neuroinflammation for weeks to months after stroke, contributing to white matter injury and compromised neurovascular unit integrity. These sustained pathological changes may ultimately lead to secondary neurodegeneration and cognitive impairment. Liu et al. (2024b) showed that circulating HMGB1 levels measured within 24 hours after acute IS can independently predict the risk of cognitive impairment at 3 months. Even after adjusting for confounders such as age and sex, the HMGB1 concentration remained significantly associated with cognitive deficits, supporting its role as a reliable predictor of PSCI.

#### Glial fibrillary acidic protein

Glial fibrillary acidic protein (GFAP) is an intermediate filament cytoskeletal protein that is specifically expressed in astrocytes. When astrocytes are damaged or activated, they secrete large amounts of this protein. Increasing evidence supports the potential clinical value of blood GFAP levels in neurodegenerative diseases such as head trauma, multiple sclerosis, cerebral hemorrhage, and IS, as well as neuroinflammation (Kraljević et al., 2024). In addition, elevated GFAP concentrations are associated with the presence of depression after IS, suggesting that neuroinflammatory responses may be related to poststroke complications (Shan et al., 2023). It has predictive value for the progression of AD and the risk of cognitive impairment (Shen et al., 2023). Shan et al. (2025) revealed that elevated serum GFAP levels during the acute phase are significantly associated with the incidence of PSCI at 90 days after stroke.

#### Soluble triggering receptor expressed on myeloid cells 2

Triggering receptor expressed on myeloid cells 2 (TREM2) is a single-pass transmembrane protein that is predominantly expressed on the surface of myeloid cells, including microglia, macrophages, and dendritic cells. Soluble TREM2 (sTREM2) is produced by the proteolytic cleavage of the extracellular domain of TREM2 by disintegrin and metalloproteinase family proteins and is subsequently released into the extracellular microenvironment. A small-sample clinical study suggested that elevated plasma sTREM2 levels during the acute phase of stroke may predict poor functional recovery (Kwon et al., 2020). Zhu et al. (2022) identified a positive dose‒response relationship between the plasma sTREM2 concentration and the risk of PSCI, indicating its potential utility as an early warning biomarker (**Additional Table 2**).

**Additional Table 2:** Summary of inflammatory biomarkers of poststroke cognitive impairment

Reference	Adjust age and gender	Assessment of poststroke cognitive impairment	Biomarker	Strengths	Limitations	Significance
Sandvig et al., 2023	Yes	Montreal Cognitive Assessment	Interleukin-6	Large prospective multicenter cohort with long-term follow-up	Observational design prevents causal inference	Predicts 3, 18, 36 months poststroke cognitive impairment
Irimie et al., 2018	Yes	National Institutes of Health Stroke Scale Mini-Mental State Examination	C-reactive protein	Uses standardized clinical scales	Small sample size; Single time-point measurement	Predicts acute phase poststroke cognitive impairment
Zhang et al., 2021	Yes	Montreal Cognitive Assessment National Institutes of Health Stroke Scale	Serum amyloid A	Prospective design with a clear 3-month follow-up for cognitive assessment.	Single-center study with a relatively small sample size; Measured only once at baseline	Predicts 3 months poststroke cognitive impairment
Shang et al., 2022	Yes	National Institutes of Health Stroke Scale Montreal Cognitive Assessment	Neutrophil-to-lymphocyte ratio	Large sample size; Demonstrates a dose-response relationship	Single-center retrospective design	Predicts 3 months poststroke cognitive impairment
Bao et al., 2023	Yes	National Institutes of Health Stroke Scale Montreal Cognitive Assessment	Systemic immune-inflammation index	Prospective design with a clear 3-month follow-up for cognitive assessment	Single-center study with a moderate sample size; Measured only once at baseline	Predicts 3 months poststroke cognitive impairment
Chu et al., 2024	Yes	National Institutes of Health Stroke Scale	Systemic inflammatory response index	Large sample size	Short-term cognitive assessment; Cross-sectional design limits causal inference	Predicts acute phase poststroke cognitive impairment
Liu et al., 2024b	Yes	National Institutes of Health Stroke Scale Montreal Cognitive Assessment	High mobility group box 1	Standardized Measurement	Small sample size	Predicts 3 months poststroke cognitive impairment
Zhu et al., 2022	Yes	National Institutes of Health Stroke Scale Mini-Mental State Examination	Soluble triggering receptor expressed on myeloid cells 2	Large sample size; Demonstrates a clear dose-response relationship between	Lacks baseline cognitive assessment; Excluded severe cases	Not described
Xu et al., 2023d	Yes	Mini-Mental State Examination	Hemoglobin, albumin, lymphocyte, and platelet	Large sample size; Uses clinically routine blood parameters	Lacks baseline cognitive assessment; Dynamic changes were not captured	Predicts acute phase poststroke cognitive impairment
Xu et al., 2022	Yes	Mini-Mental State Examination	Lactate dehydrogenase-to-albumin ratio	First study to explore lactate dehydrogenase-to-albumin ratio and poststroke cognitive impairment	Single-center study; Cognitive assessment limited to the acute phase	Predicts acute phase poststroke cognitive impairment

### Genetic biomarkers

#### miR-132, miR-let-7i, miR-195, and miR-497

MicroRNAs (miRNAs) are a class of noncoding RNA molecules that regulate gene expression through post transcriptional mechanisms. A previous study has detected miRNAs in serum and linked specific circulating miRNA profiles to various diseases, including cardiovascular disorders, stroke, and multiple sclerosis. miR-132 plays a key role in regulating neuronal morphology and synaptic function (Ma et al., 2022). Dysregulation of miR-132 expression in the mature nervous system can disrupt synaptic protein homeostasis, leading to synaptic dysfunction and subsequent cognitive impairment. Huang et al. (2016) demonstrated that compared with those in non-PSCI patients, miR-132 levels in patients with PSCI were significantly elevated, with an AUC of 0.961. Similarly, Wang et al. (2020) observed that serum miR-let-7i expression was significantly upregulated in the PSCI group and strongly negatively correlated with the MoCA score. Additionally, Zhai et al. (2020) reported that serum levels of miR-195 and miR-497 in patients with acute stroke have high diagnostic value for cognitive impairment.

#### Hypomethylation of Ras and Rab interactor 3 and methylation of matrix metalloproteinases-9

DNA methylation is a key mechanism involved in the pathogenesis of several neurodegenerative disorders, including AD, PD, and stroke. Ras and Rab interactor 3 (RIN3), located on chromosome 14, acts as a stimulatory factor to stabilize the transport of GTP-RAB5 to the plasma membrane and endosomes. This process negatively affects the endocytosis of Aβ. Miao et al. (2021) reported that patients with early cognitive impairment following TIA or mild IS exhibited significantly lower methylation levels in the RIN3 gene promoter region than cognitively normal individuals. Concurrently, Aβ accumulation was observed in patients with TIA or mild IS. As a member of the gelatinase family, MMP-9 is markedly upregulated during the acute phase of IS and can compromise BBB integrity by degrading type IV collagen and laminin. Elevated serum MMP-9 levels in IS patients are associated with a poor prognosis (Zhong et al., 2017). Furthermore, Miao et al. (2023) found that among patients with TIA/mild IS, those with early cognitive impairment had lower methylation levels of the MMP-9 gene than those without cognitive impairment.

#### Brain-derived neurotrophic factor Val66Met gene

Accumulating evidence strongly indicates that the BDNF Val66Met polymorphism (rs6265), a key functional genetic variant, significantly impairs the biological activity of the BDNF protein through a valine-to-methionine substitution at codon 66. The relationship between the BDNF Val66Met polymorphism and PSCI has attracted considerable research interest. A previous study has confirmed that the BDNF Val66Met polymorphism not only increases the risk of PSCI but also contributes to deficits across multiple cognitive domains (Sadhukhan et al., 2023). Han et al. (2020) demonstrated that stroke patients carrying the BDNF Met allele exhibited significantly poorer cognitive function at discharge after ischemic or hemorrhagic stroke compared to those who do not carry the allele. Notably, the BDNF Met allele occurs more frequently in patients with cognitive impairment, whereas the Val/Val genotype is less common in this population. Rezaei et al. (2022) found that individuals carrying the BDNF Met/Met homozygous genotype experienced a mitigation of the cognitive impact of dominant hemisphere lesions, brain atrophy, and multifocal lesions. In contrast, carriers of the Val allele (Val/Met heterozygotes) exhibited more severe cognitive impairment when such lesions were present (**Additional Table 3**).

**Additional Table 3:** Summary of genetic biomarkers of poststroke cognitive impairment

Reference	Adjust age and gender	Assessment of poststroke cognitive impairment	Biomarker	Strengths	Limitations	Significance
Huang et al., 2016	Yes	Montreal Cognitive Assessment	microRNA-132	First study to link serum microRNA-132 levels with post-stroke cognitive impairment	Small sample size; Single time-point measurement	Predicts 1 year poststroke cognitive impairment
Wang et al., 2020	Yes	Montreal Cognitive Assessment	microRNA-let-7i	First study to identify microRNA-let-7i as a potential biomarker	Small sample size; Lack of longitudinal data to track microRNA-let-7i changes over time	Predicts 1 year poststroke cognitive impairment
Zhai et al., 2020	Not described	Montreal Cognitive Assessment National Institutes of Health Stroke Scale	microRNA-195 microRNA-497	Evaluated two miRNAs (microRNA-195 and microRNA-497) simultaneously	Single-center design may limit generalizability; Did not adjust for all potential confounders	Predicts acute phase poststroke cognitive impairment
Miao et al., 2021	Yes	National Institutes of Health Stroke Scale	Hypomethylation of RIN3	First study RIN3 promoter hypomethylation and cognitive	Small sample size; Cross-sectional design limits causal inference	Predicts acute phase poststroke cognitive impairment
Miao et al., 2023	Yes	National Institutes of Health Stroke Scale	Methylation of the matrix metalloproteinases-9	First study methylation of the matrix metalloproteinases-9 and cognitive	Small sample size	Predicts acute phase poststroke cognitive impairment
Rezaei et al., 2022	Not described	Mini-Mental State Examination	Brain-derived neurotrophic factor Val66Met Gene	Uses comprehensive neuroimaging	Small sample size	Predicts 2 years poststroke cognitive impairment

RIN3: Ras interacting protein 3.

### Biomarkers of blood–brain barrier disruption

#### Amyloid-β42 and p-tau181

Amyloid-β42 (Aβ_42_), a neurotoxic peptide derived from residues 672–711 of the amyloid precursor protein (APP), has a strong tendency to aggregate and form amyloid plaques. Xu et al. (2023d) observed a significant decrease in the Aβ_42_ concentration in patients with PSCI compared with controls. The combination of Aβ_42_ and Hb improved diagnostic accuracy, achieving an AUC of 0.7169. In contrast, Shi et al. (2024) reported significantly higher Aβ_42_ levels in patients with cerebral infarction and cognitive impairment than in controls. Additionally, Mao et al. (2020) found that serum Aβ_42_ levels were significantly lower in the PSCI group than in the non-PSCI group and that the combined detection of Aβ_42_ and T3 substantially improved the predictive performance. A pathological study has shown that hyperphosphorylated tau proteins self-assemble into paired helical filaments, which further aggregate to form neurofibrillary tangles, a hallmark neuropathological feature of AD (Moore et al., 2023). Huang et al. (2022) conducted a study of 136 patients with acute IS and observed elevated plasma p-tau181 levels during the acute phase, which correlated with a reduced risk of PSCI at both 3 and 12 months. Their longitudinal cohort analysis revealed a dose-dependent relationship, with the highest p-tau181 levels detected in the persistent non-PSCI group and the lowest in the persistent PSCI group. Additionally, Huang et al. (2021a) reported that both Aβ and tau are associated with cognitive impairment three months after stroke.

#### Matrix metalloproteinases-9 and tissue inhibitor of metalloproteinase-1

MMPs are a family of zinc- and calcium-dependent neutral proteases that regulate extracellular matrix degradation across various tissues. Following cerebral ischemia, multiple cell types within the CNS, including astrocytes, microglia, macrophages, infiltrating neutrophils, and ECs, can produce MMP-9 through both endogenous and exogenous pathways. MMP-9 degrades the basement membrane surrounding cerebral blood vessels by cleaving type IV and V collagen, thereby compromising the integrity of the BBB. Tissue inhibitor of metalloproteinase-1 (TIMP-1), an endogenous inhibitor of MMP-9, participates in various pathophysiological processes by regulating extracellular matrix degradation, inflammatory responses, fibrotic progression, apoptosis and differentiation, and angiogenesis. Following acute cerebral ischemia, the concentration of TIMP-1 increases in the peripheral blood of patients, and its dynamic changes are significantly correlated with the prognosis of IS. Pu et al. (2022) found that the serum concentrations of both MMP-9 and TIMP-1 were significantly higher in patients with PSCI upon admission than in healthy controls. The combined detection of MMP-9 and TIMP-1 substantially improved the accuracy of predicting the PSCI prognosis at 3 months. Similarly, Ge et al. (2020) demonstrated that increased levels of both TIMP-1 and MMP-9 are associated with a significantly increased risk of cognitive impairment (**Additional Table 4**).

**Additional Table 4:** Summary of blood-brain barrier disruption biomarkers of poststroke cognitive impairment

Reference	Adjust age and gender	Assessment of poststroke cognitive impairment	Biomarker	Strengths	Limitations	Significance
Mao et al., 2020	Yes	National Institutes of Health Stroke Scale	Amyloid-β42 Triiodothyronine	Uses repeated measures analysis of variance to show temporal trends in biomarker levels	Small sample size; Excluded severe cases	Predicts lyear poststroke cognitive impairment
Huang et al., 2022	Not described	National Institutes of Health Stroke Scale	Phosphorylated tau protein	Identified plasma p-tau181 as a strong protective predictor	Small sample size; No pre-stroke cognitive assessment	Predicts early- and delayed phase poststroke cognitive impairment
Ge et al., 2020	Yes	Mini-Mental State Examination Montreal Cognitive Assessment	Matrix metalloproteinases-9 Tissue inhibitor metalloproteinase-1	Use oftwo validated cognitive assessment tools; Explored joint effects of matrix metalloproteinases-9 And tissue inhibitor metalloproteinase-1	Single-timepoint measurement	Predicts 3 months poststroke cognitive impairment

### Biomarkers of gut microbiomics

#### Firmicutes, proteobacteria, enterococcus, Bacteroides, and Escherichia–shigella

In recent years, the “gut microbiota–brain axis” has emerged as a prominent focus in research on stroke prevention and treatment. A previous study has established a strong association between the gut microbiomics (GM) and the incidence, progression, and prognosis of stroke (Peh et al., 2022). Xu et al. (2018) reported that the GM profile of patients with acute IS was characterized by elevated abundances of *Bifidobacterium*, *Macromonas*, *Brucella*, *Haldeman*, and *Clostridium*. In 2020, Liu et al. (2020) proposed a correlation among PSCI, the GM, and metabolites. Li et al. (2022a) found that compared with the other groups, the PSCI group had significantly higher levels of *Bifidobacterium*, *Alloscardovia*, and *Alloscardoviaomnicolens* (Actinobacteria phylum), *Anaerostipeshadrus* and *Lactobacillus gasseri* (Firmicutes phylum). Feng et al. (2021) reported that compared with healthy controls, PSCI patients presented a decrease in the abundance of *Firmicutes* and an increase in the abundance of *Proteobacteria*. In addition, the differences in bacterial species between the groups correlated with cognitive function, and these species are expected to become targets for the early screening and intervention of PSCI. Ling et al. (2020) found that the relative abundance of Proteobacteria in the GM of patients in the PSCI group was significantly higher than that in the non-PSCI group. In another study, the abundances of *Enterococcus*, *Bacteroidetes*, and *Escherichia*–*Shigella* in the GM of patients with PSCI was significantly higher than that in the GM of patients with stroke alone (Huang et al., 2021c). Wu et al. (2025) reported increased abundances of the genera *Blautia*, *Bifidobacterium*, and *Megabacter* in patients with PSCI, whereas the abundances of the genus *Bacteroides* and species *Bifidobacterium brevis* were significantly reduced. In addition, Jeng et al. (2025) reported that compared with non-PSCI patients, PSCI patients had an increased abundance of *Bacteroidaceae* in their intestines, whereas the abundances of *Ruminococcaceae* and *Prevotella* decreased.

### Imaging biomarkers

#### Medial temporal lobe atrophy, white matter hyperintensity, cortical superficial siderosis, leukoaraiosis, hippocampal texture features, and cortical atrophy

**Figure 4:**
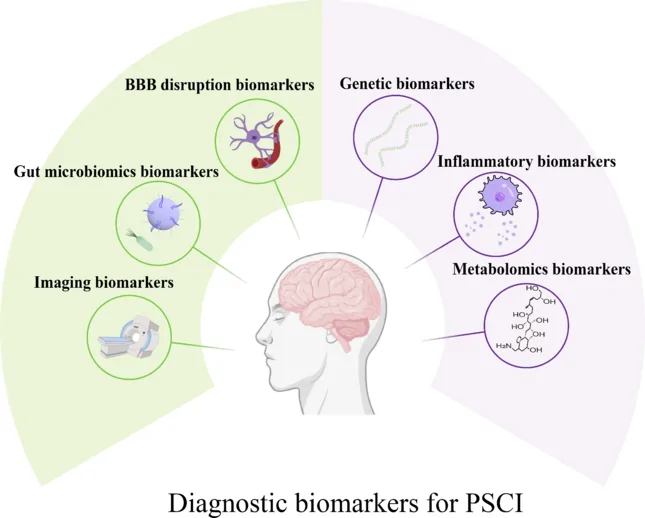
4 Overview of PSCI biomarkers. This figure systematically summarizes the multidimensional biomarker research framework for assessing and predicting post-stroke cognitive impairment, covering multi-omics levels including genetics, gut microbiome, imaging, and metabolomics. BBB: Blood–brain barrier; PSCI: poststroke cognitive impairment.

Recent advances in neuroimaging techniques have significantly enhanced the utility of MRI in predicting and evaluating post-stroke cognitive impairment (PSCI). A previous study indicated that MRI-detected structural alterations in specific brain regions are strongly associated with the development of PSCI (Dragoș et al., 2025). Medial temporal lobe atrophy (MTLA), a key imaging biomarker for neurodegenerative and cerebrovascular cognitive disorders, has garnered considerable attention from researchers studying PSCI. Casolla et al. (2019) confirmed a significant correlation between MTLA and the progression of PSCI, identifying both whole-brain atrophy and MTLA as the most consistent and reliable neuroimaging predictors. A 7-year prospective cohort study conducted by Hagberg’s team revealed that patients with a younger baseline age and milder MTLA, as assessed by standardized MRI grading, showed improved cognitive outcomes at the 12-month follow-up (Hagberg et al., 2019). Notably, Wang et al. (2021a) innovatively demonstrated that combining MTLA with WMH, a hallmark of cerebral small vessel disease, significantly enhances the accuracy of predicting the risk of PSCI. Ali et al. (2023) showed that severe WMH not only significantly increases the risk of cognitive decline but may also contribute to the recurrence of post-stroke vascular events and is associated with more severe cognitive impairment 3 years later. Sagnier et al. (2021) further demonstrated that the severity of cerebral small vessel disease biomarkers, including WMH and cortical superficial siderosis, is negatively correlated with recovery from PSCI. Tziaka et al. (2023) confirmed a significant association between leukoaraiosis and PSCI, noting that the severity of leukoaraiosis exhibits a clear dose-response relationship with impairments in specific cognitive domains, such as executive function, attention, and information processing speed. Additionally, structural alterations in the hippocampus have been closely linked to PSCI. Studies in both human and animal models have reported hippocampal deformation and atrophy in olfactory-related cortical regions six months after a stroke. In T1-weighted MRI scans performed within 72 hours post stroke, Betrouni et al. (2020) identified that specific texture features, such as reduced kurtosis and inverse difference moment in the hippocampus and entorhinal cortex, can predict cognitive decline at 6 months. Together, these findings provide crucial imaging evidence to support early identification and intervention for PSCI. Furthermore, current research has indicated that stroke can lead to both cortical and subcortical atrophy. Aamodt et al. (2022) observed more pronounced cortical atrophy in the affected hemisphere among stroke patients compared to controls. Notably, their study also revealed that atrophy in these regions was significantly associated with persistent cognitive decline. Thus, post-stroke cortical thickness is clearly correlated with PSCI and should be incorporated into PSCI risk assessment frameworks (**Figure 4**).

### Electrophysiological biomarkers

Electroencephalography (EEG) is a low-cost, noninvasive neuroimaging technique that is widely used in clinical diagnosis and treatment. Owing to its high temporal resolution, EEG can directly capture dynamic brain activity and detect subtle pathophysiological processes. A recent study has indicated that quantitative EEG measures are sensitive to cognitive impairment following stroke (Chai et al., 2023). Hadiyoso et al. (2023) and Hong et al. (2022) reported that compared with healthy individuals, patients with mild PSCI exhibit enhanced δ waves and weakened α and β waves in terms of their EEG characteristics. Xu et al. (2024b) revealed that in patients with severe PSCI, increased θ and α power was detected in the right frontal lobe region, whereas in patients with mild to moderate PSCI, increased θ and β power was detected in the parietal lobe region. Notably, abnormal γ power is related to the severity of the disease (Braverman-Jaiven et al., 2022). In addition, at the functional connectivity level, patients with severe PSCI exhibit overall increased connectivity, which may be a pathological compensatory response (Ruan et al., 2025). Petrovic et al. (2017) found that during the subacute phase, abnormal α rhythms, including a slowed α frequency, decreased frontal lobe phase potential, and disrupted α flow patterns, were observed in patients with right middle cerebral artery stroke. In summary, these findings indicate that EEG spectral features objectively reflect the neural mechanisms of PSCI and can serve as potential biomarkers for its evaluation.

## Clinical Translation

Cognitive impairment is a frequent complication following stroke, which not only prolongs the rehabilitation process but can also adversely affect the patient’s mental state and physical function. Clinical studies have shown that the first 3 months after a stroke constitute a critical period for functional recovery (Corrigan et al., 2024). DL-3-n-butylphthalide (NBP) is a novel drug developed in China, derived from the natural active compound L-3-n-butylphthalide, which was originally isolated from celery seeds. This synthetic agent has demonstrated distinctive clinical value in the treatment of cerebrovascular diseases, improving acute symptoms of IS and supporting long-term functional recovery in patients. Extensive preclinical studies have confirmed the multitarget protective effects of NBP in animal models of IS (Wang et al., 2023a), with mechanisms involving multiple pathophysiological processes, including anti-inflammatory activity, antioxidant activity (Gao et al., 2022), reduction of cerebral edema, preservation of the BBB (Ma et al., 2023), inhibition of neuronal apoptosis (Zhang et al., 2022a), and prevention of thrombosis (Yu et al., 2024). A recent study has shown that NBP can promote cognitive function recovery after stroke, with mechanisms that may involve the regulation of the inflammatory response and oxidative stress (Zhang et al., 2022a). The expert consensus on PSCI management in 2021 lists NBP as a therapeutic drug for PSCI (Class II, Level B evidence) (Quinn et al., 2021). Furthermore, Fan et al. (2021) substantiated that NBP treatment can significantly improve the neurological deficits of PSCI patients, enhancing their cognitive ability and daily self-care levels. An ongoing clinical trial (NCT05976152) is systematically evaluating the impact of NBP on cognitive function following cerebrovascular events (**Box 1**). Wang et al. (2024c) found that the cognitive improvement associated with NBP can be observed within 1 week of treatment and can last for up to 6 months, confirming that the drug has a rapid onset and prolonged therapeutic effect. Notably, NBP treatment reduced the levels of pro-inflammatory and injury markers in PSCI patients while increasing the expression of neurotrophic factors (BDNF and VEGF). These comprehensive changes in biomarkers provide objective evidence for understanding the therapeutic mechanisms of NBP.

Box 1: North American clinical trial registry program (NCT05976152; 2024)

**(1) Significance:** To evaluate the efficacy of donepezil in treating PSCI and to address the gap in large randomized controlled trials within this field.

**(2) Methodology:** A multicenter, randomized, double-blind, placebo-controlled trial conducted in two phases (0–24 weeks assessing preventive effects, 24–48 weeks assessing therapeutic effects).

**(3) Insights for the paper:** It provides high-level evidence for drug therapy in PSCI, with its two-phase design serving as a methodological reference for staged intervention research in cognitive impairment.

**(4) Safety assessments:** NBP: Listed as a treatment drug for PSCI (Class II recommendation, Level B evidence).

**FDA’s new drug approval status and the current status of clinical translation**

No new drugs have yet been approved by the FDA for PSCI.

## Limitations

This review comprehensively summarizes the current evidence on the pathological mechanisms and biomarkers related to PSCI, yet several important limitations must be acknowledged. Currently, PSCI research is largely limited to observational studies or small-sample clinical trials and lacks large-sample multicenter prospective studies or RCTs, which affects the generalizability and prospective clinical application of the research findings. Furthermore, substantial heterogeneity exists in biomarker detection methodologies across studies due to variations in sample collection, processing methods, and analytical techniques, making cross-study comparisons and data integration challenging. The dynamic changes in biomarkers and their causal relationships with PSCI progression remain unclear, particularly regarding long-term correlations with the evolution of cognitive function. From a technical perspective, while noninvasive neuroimaging techniques and electroencephalography provide valuable insights into structural and functional brain abnormalities as well as neural network dysfunction, their clinical application lacks standardization in diagnostic thresholds, scanning protocols, and data analysis methods. Regarding the pathological mechanisms, current hypotheses, including neuroinflammation, BBB disruption, impaired synaptic plasticity, and white matter damage, remain insufficiently supported by direct molecular or cellular biological evidence.

## Discussion

The “Expert Consensus on the Management of Post-Stroke Cognitive Impairment” states that the occurrence of PSCI is closely related to nonmodifiable factors such as sex, age, ethnicity, genetics, and education level, as well as modifiable factors such as hypertension, diabetes, stroke history, obesity, and unhealthy lifestyles (Dong et al., 2017; Wang et al., 2021c). A clinical diagnosis requires a combination of clinical presentation, imaging studies, and neuropsychological assessments, and a definitive diagnosis can be made only 3 months after the stroke event. Therefore, identifying biomarkers with high specificity and sensitivity has become a current research priority. The concept of a “vascular cognitive impairment biomarker spectrum,” proposed at the 2022 International Stroke Conference, further highlights the importance of biomarker research. However, current studies on biomarkers are limited by their focus on single biomarkers. This review provides a detailed explanation of the pathophysiological mechanisms of PSCI and its associated biomarker profiles, recommending that clinicians conduct comprehensive assessments by combining neuropsychological scale scores, blood biomarker testing, and imaging characteristics. Notably, establishing a multi-indicator predictive model (including miRNA profiles, inflammatory factors (Rothenburg et al., 2010), and other blood markers, as well as white matter lesions, cortical thickness, and other imaging parameters) can significantly improve diagnostic accuracy.

Current research has revealed close relationships between PSCI and GM and inflammatory markers. The GM can exert extensive regulatory effects on the nervous, metabolic, and immune systems through the gut–brain axis (Angoorani et al., 2022). Notably, the dysbiosis of specific pathogenic microorganisms (such as *Chlamydia pneumoniae*, *Helicobacter pylori*, and *Porphyromonas gingivalis*) can significantly increase CRP levels, thereby promoting the development of atherosclerosis and IS. Another study found that patients who underwent the Montreal Cognitive Assessment 3 months after stroke and were diagnosed with PSCI had a significantly higher abundance of *Enterobacteriaceae bacteria* in the gut and higher levels of IL-6 and IL-1β in plasma than those without PSCI (Wang et al., 2022b). A mechanistic study has shown that post stroke *Enterobacteriaceae* can release lipopolysaccharides and enter the brain parenchyma through the damaged BBB, activating inflammatory pathways and upregulating the expression of pro-inflammatory factors such as IL-6. A longitudinal study has shown that a decrease in the abundance of these bacteria is significantly associated with elevated p-tau181 levels and disease progression in patients with MCI (Yang et al., 2023). These findings collectively suggest that the GM-inflammation network may be involved in the pathological process of PSCI through multiple mechanisms. Additionally, a correlation has been identified between Aβ and tau levels in plasma and neuroimaging biomarkers. Huang et al. (2021a) found that plasma tau and tau*Aβ_42_ levels were correlated with mean cortical thickness after adjusting for age. A decrease in the Aβ_42_/Aβ_40_ ratio may indicate the accumulation of amyloid plaques in the brain. Furthermore, Kandiah et al. (2016) developed a clinical and imaging-based risk scoring system. Their results indicated that a 15-point risk score based on age, education level, acute cortical infarction, WMH, chronic lacunar infarcts, global cortical atrophy, and intracranial large vessel stenosis highly predicted PSCI.

This review also explores the mechanisms underlying PSCI. Neuroinflammatory activation and oxidative stress are the core driving mechanisms, while post-stroke white matter damage, BBB disruption, cerebellar infarction, and impaired synaptic plasticity may collectively contribute to the pathogenesis of PSCI. Based on in-depth investigations into the pathophysiological mechanisms of PSCI, miR-132 and BDNF have been identified as key therapeutic targets for regulating cognitive function and promoting neural recovery. Building on this foundation, we further propose a dynamic biomarker monitoring system and have implemented a risk-stratified management strategy: high-risk patients require comprehensive cognitive function and biomarker assessments every 3 months, whereas moderate-to-low-risk patients are monitored according to a stepwise protocol. This integrated management framework enables early prediction 6 to 12 months before clinical manifestations appear, providing critical support for precise intervention in PSCI.

## Additional files:

***Additional Table 1:*** *Summary of metabolic biomarkers of poststroke cognitive impairmemt.*

***Additional Table 2:*** *Summary of inflammatory biomarkers of poststroke cognitive impairmemt.*

***Additional Table 3:*** *Summary of genetic biomarkers ofpoststroke cognitive impairmemt.*

***Additional Table 4:*** *Summary of blood–brain barrier disruption biomarkers of poststroke cognitive impairmemt.*

## Data Availability

*All relevant data are within the paper and its Additional files.*
